# Fluorodeoxyglucose Positron Emission Tomography integrated with computed tomography in carcinoma of the cervix: Its impact on accurate staging and the predictive role of its metabolic parameters

**DOI:** 10.1371/journal.pone.0215412

**Published:** 2019-04-18

**Authors:** Ismaheel O. Lawal, Thabo Lengana, Charl Janse van Rensburg, Florette Reyneke, Gbenga O. Popoola, Alfred O. Ankrah, Mike M. Sathekge

**Affiliations:** 1 Department of Nuclear Medicine, University of Pretoria, Pretoria, South Africa; 2 Biostatistics Unit, South African Medical Research Council, Pretoria, South Africa; 3 Department of Epidemiology and Community Health, University of Ilorin, Ilorin, Nigeria; 4 Department of Nuclear Medicine and Molecular Imaging, University Medical Center, Groningen, The Netherlands; Ente Ospedaliero Cantonale, SWITZERLAND

## Abstract

**Objectives:**

To determine the impact of FDG-PET/CT in the initial staging of cervical cancer among women with and without HIV and to determine the abilities of FDG-PET/CT metabolic parameters in predicting the presence of distant metastasis.

**Methods:**

We reviewed the FDG-PET/CT images of women with FIGO stage IB2 to IVA carcinoma of the cervix. We compared the FIGO stage before and after FDG-PET/CT. Maximum and mean standardized uptake values (SUVmax and SUVmean), metabolic tumor volume (MTV) and total lesion glycolysis (TLG) of the primary lesion were determined. We compared these parameters between the HIV-infected and uninfected woman and also determined their abilities to predict the presence of distant metastasis.

**Results:**

126 women, mean age 48.05 ± 11.80 years were studied. Seventy-three patients were HIV-infected. The disease was upstaged in 65 patients, 32 of which were upstaged to stage IVB. HIV-infected women were younger (43.36 ± 8.03 years versus 54.51 ± 13.12, *p*<0.001) and had more advanced disease (*p* = 0.022) compared with HIV-uninfected. In a univariate logistic regression adjusted for the FIGO stage of the disease, there were significant associations between MTV and TLG of the primary tumor and distant metastasis. SUVmax, SUVmean, MTV and TLG performed well in predicting the presence of distant metastasis with areas under the curves (AUCs) of 0.63, 0.66, 0.80 and 0.77 respectively. These performances improved after adjustment for the FIGO stage of the disease with AUCs of 0.80, 0.79, 0.84 and 0.82 for SUVmax, SUVmean, MTV and TLG respectively.

**Conclusion:**

Inclusion of ^18^F-FDG-PET/CT in the pre-therapy assessment of cervical cancer improves the accuracy of staging in about half of the patients. The metabolic parameters of the primary tumor perform well in predicting the presence of distant metastases.

## Introduction

Carcinoma of the cervix is one of the most common gynecological malignancies and a significant cause of cancer mortality worldwide [1–3]. Accurate staging is a necessary prerequisite to determine the most appropriate therapeutic option. The staging of disease is done according to the International Federation of Gynecology and Obstetrics (FIGO) recommendations [4]. FIGO staging entails a thorough pelvic examination, colposcopy, endocervical curettage, hysteroscopy, cystoscopy, proctoscopy, intravenous urography and radiological evaluation of the lungs and skeleton for metastasis.

Hybrid positron emission tomography integrated with computed tomography using fluorodeoxyglucose (FDG-PET/CT) is an integrated metabolic and morphologic modality for oncologic imaging that outperforms stand-alone morphologic imaging. Metabolic parameters such as maximum standardized uptake value (SUVmax), metabolic tumor volume (MTV) and total lesion glycolysis (TLG) derivable from FDG-PET/CT reflect the tumor metabolism and its aggressiveness. These metabolic parameters have been shown to predict survival in different malignancies [5]. Despite its excellent ability to detect the site of unknown metastasis in patients with bulky primary tumor, FDG-PET/CT is still not routinely used for initial staging of patients with carcinoma of the cervix. Lack of accurate staging of patients before the institution of therapy may result in ineffective treatment with its attendant morbidity and cost.

Carcinoma of the cervix has a strong association with persistent infection with high-risk strains of human papillomavirus (HPV) infection, an oncogenic virus that predisposes to high-grade intraepithelial lesions [6]. Individuals with human immunodeficiency virus (HIV) infection have a higher prevalence of high-grade intraepithelial lesion and consequently invasive squamous cell carcinoma (SCC) of the cervix [7,8]. Studies have shown the utility of FDG-PET metabolic parameters in predicting distant metastasis as well as patients’ survival [9–11]. Most of these studies were done in countries with a low incidence of HIV infection. It remains unknown if findings from these studies can be translated to HIV-associated invasive carcinoma of the cervix. The aim of this study was, therefore, to determine the impact of FDG-PET/CT in the initial staging of a mixed population of women with and without HIV infection diagnosed with carcinoma of the cervix as well as determine the abilities of FDG-PET/CT metabolic parameters in predicting the presence of distant metastasis.

## Materials and methods

### Patients

We reviewed the FDG-PET/CT scan images of 519 consecutive patients with histological diagnosis of carcinoma of the cervix who were referred to the Department of Nuclear Medicine at Steve Biko Academic Hospital in Pretoria from January 2014 to March 2018. Steve Biko Academic Hospital is one of the largest academic hospitals in South Africa that serve as a referral center from most areas of two of the nine provinces of the country. The hospital also receives referrals from other neighboring Southern African countries. We excluded patients who obtained FDG-PET/CT scan for indications other than initial staging, those with FIGO stages IA, IB1 and IVB disease, those in with vesicovaginal fistula complicating their disease and technically sub-optimal scans. We reviewed the medical records of patients who met our inclusion criteria to extract the following information: epidemiological parameters, FIGO stage before FDG-PET imaging, histological sub-type of the tumor, and HIV status. In patients with HIV infection, we extracted the CD 4 count and viral load. In our institution, patients who are willing to allow their FDG-PET/CT imaging data to be used anonymously for research purpose sign a consent form. We only used data from patients who had previously signed this consent form in this study. The institutional review board of the University of Pretoria approved this study (Reference No:223/2018).

### FDG-PET/CT Imaging

A standard patient preparation was observed. Each patient fasted for a minimum of six hours and fasting blood sugar was less than 11 mmol/L at the time of FDG injection. FDG was injected intravenously with activity adjusted for patients’ weights. PET/CT imaging commenced 60 minutes following FDG administration. We performed imaging on a Biograph 40 Truepoint hybrid PET/CT scanner (Siemens Medical Solution, Illinois, USA). Where no contraindication existed, we imaged the patients with the administration of oral (Gastrografin, Bayers, Isando, South Africa) and intravenous (Omnipaque, GE Healthcare, Wisconsin, USA) contrast. We acquired PET images in 3D mode at 3 minutes per bed position in a caudocranial direction. We performed PET image reconstruction using ordered subset expectation maximization iterative reconstruction (4 iterations, eight subsets) with a Gaussian filter applied at full-width at half-maximum of 5.0mm.

### Image analysis and interpretation

We performed image analysis and interpretation on a dedicated workstation equipped with a Syngo.Via software (Siemens Medical Solution, Illinois, USA). We drew a semi-automatic spherical volume of interest (VOI) around the primary cervical lesion while taking care to exclude bladder, ureters and other areas of increased physiologic or pathologic FDG uptake. We used an SUV threshold of 2.5 and a 3D isocontour of 41%. We recorded the SUVmax, SUVmean, and MTV of the primary cervical lesions that were automatically computed by the software. We manually computed the TLG of the primary cervical lesion by multiplying SUVmean by the MTV.

Two nuclear medicine physicians with five years (IOL) and more than ten years (MMS) experience interpreting oncologic FDG-PET/CT images performed qualitative image interpretation to determine, by consensus, the presence, and site of distant metastasis. Nodal metastases were classified as either pelvic nodes when localized to the pelvic region or extra-pelvic nodes when localized elsewhere. We classified as visceral metastasis any metastatic lesion seen in the soft tissue visceral (such as liver, lung, brain), peritoneum and peri-umbilical nodule.

We confirmed the findings on FDG-PET/CT as metastatic by histological examination in 64 patients. Where biopsy was not possible (n = 62), the findings were correlated with magnetic resonance imaging (MRI) or based on consensus interpretation by the interpreting physicians.

We revised the FIGO staging following the confirmation of FDG-PET/CT findings. We determined the proportion of patients who had a FIGO stage migration following FDG-PET/CT.

### Statistical analysis

We expressed continuous variables as the mean ± standard deviation (SD) when they were normally distributed or as median (interquartile range, IQR) when they were skew. We expressed categorical data as proportions (percentages). We used the Chi-square test to compare categorical data, and the Mann Whitney U test and independent samples T-test for continuous variables. We used logistic regression to determine the association between the FDG-PET metabolic parameters and the presence of metastasis, first unadjusted, then adjusted for FIGO stage of disease. We constructed adjusted ROC curves of SUVmax, SUVmean, MTV, and TLG and reported the Area under the Curve (AUC). All statistical analyses were two-tailed, and p-value <0.05 was considered statistically significant. Analyses were performed using IBM SPSS Statistics 21.0 (IBM Corp, Armonk, New York USA) and STATA statistical Software version 15 (College Station, TX: StataCorp LLC).

## Results

A total of 126 women met our inclusion criteria and were included in this study, mean age = 48.05 ± 11.80 years. There were 73 patients (57.9%) with HIV infection while 53 patients (42.1%) were HIV-negative. About a half of our study population had a FIGO stage IIIB or IVA disease at the time of referral for FDG-PET/CT. We found SCC as the most common histological type of carcinoma of the cervix in our study population (87.3%). Other histological variants seen included adenocarcinoma (7.9%), adenosquamous (1.6%), neuroendocrine (1.6%) and other rare variants (1.6%). Table 1 shows the detailed baseline clinic-pathologic characteristics of the study population.

**Table 1:** Clinical and pathologic characteristics of the study population.

Variable	HIV + (n = 73)	HIV–(n = 53)	Overall (n = 126)	p-value
**Age (years)**				
Mean ± SD	43.36 ± 8.03	54.51 ± 13.12	48.05 ± 11.80	<0.001*
Range			25–82	
**FIGO stage**				
IB	19 (26.0)	8 (15.1)	27 (21.4)	0.022*
IIA	2 (2.7)	4 (7.5)	6 (4.8)	
IIB	11 (15.1)	20 (37.7)	31 (24.6)	
IIIB	38 (52.1)	19 (35.8)	57 (45.2)	
IVA	3 (4.1)	2 (3.8)	5 (4.0)	
**Histology**				
Squamous cell carcinoma	68 (93.2)	42 (79.2)	110 (87.3)	0.021*
Adenocarcinoma			10 (7.9)	
Adenosquamous			2 (1.6)	
Neuroendocrine			2 (1.6)	
Mixed squamous cell carcinoma, adenocarcinoma and neuroendocrine			1 (0.8)	
Sarcomatoid				
**CD4 count (cells/uL)**				
Median (IQR)	480.0 (330.0–616.0)		-	-
Range	49–1190			
**Viral load (copies/mL)**				
Median (IQR)	132.50 (37.3–13388.0)		-	-
Range	21.0–52115.0			

*: p-value <0.05; CD4: cluster of differentiation 4; HIV: human immunodeficiency virus; FIGO: International Federation of Gynecology and Obstetrics

### FDG-PET/CT image findings

Following FDG-PET/CT scans, regional or distant metastases were seen in 88 patients (69.8%). Among these 88 patients, metastases were localized to the pelvic nodes only in 27, to extra-pelvic nodes only in five, to both pelvic and extra-pelvic nodes in 27, and to a combination of nodes and visceral organs in 18 patients. In one patient, metastasis was identified in a visceral organ, in four patients to bone and lymph nodes, and to nodes, visceral organs, and bone in 6 patients. Table 2 shows the detailed patients’ disease findings following the FDG-PET/CT scan. Following FDG-PET/CT, 65 patients (51.6%) had a FIGO stage migration to a higher stage (upstaged). Of 65 patients upstaged, 32 of them (49.2%) were upstaged to stage IVB. Twenty-one of the patients upstaged to FIGO stage IVB were staged IIIB before F-FDG-PET/CT, nine were from stage IIB and one each from stage 1B and stage IVA. In 25 patients, disease was upstaged to stage IVA. Invasion into the bladder or rectum was confirmed with MRI in 18 patients. The remaining patients proceeded to treatment based on FDG PET/CT findings without further testing. No patient was down-staged following FDG-PET/CT imaging. Table 3 shows details of stage migration in 65 patients that were upstaged.

**Table 2:** Disease characteristics of patients following FDG-PET/CT imaging.

Variable	HIV+	HIV-	Total n (%)	p-value
**Metastasis**				
Yes	51 (69.9)	37 (69.8)	88 (69.8)	0.995
No	22 (30.1)	16 (30.2)	38(30.2	
**Site of metastasis (n = 88)**				
Pelvic node only	18 (35.3)	9 (24.3)	27(30.7	0.404
Extrapelvic node only	2 (3.9)	3 (8.1)	5(5.7	
Pelvic and extra-pelvic node	14 (27.5)	13 (35.1)	27(30.7	
Visceral only	1 (2.0)	0 (0.0)	1(1.1	
Node and visceral	9 (17.6)	9 (24.3)	18(20.5	
Node and bone	4 (7.8)	0 (0.0)	4(4.5	
Node, visceral and bone	3 (5.9)	3 (8.1)	6(6.8	
**Upstage Post PET**				
Yes	36 (49.3)	29 (54.7)	65(51.6)	0.549
No	37 (50.7)	24 (45.3)	61(48.4)	
**SUV max**				
Median (IQR)	18.66 (10.88–26.47)	21.12 (13.07–26.28)	19.5 (11.6–26.4)	0.194
Range			2.7–80.4	
**SUV mean**				
Median (IQR)	5.70 (4.26–7.35)	6.20 (4.48–7.65)	5.9 (4.3–7.5)	0.310
Range			1.9–18.8	
**MTV**				
Median (IQR)	224.35 (71.81–367.36)	177.91 (111.12–268.98)	188.8 (83.9–317.6)	0.588
Range			10.8–975.6	
**TLG**				
Median (IQR)	1355.97 (297.60–2700.47)	1164.06 (543.57–1921.49)	1227.9 (424.6–2376.7)	0.974
Range			29.9–8672.6	

PET: Positron Emission Tomography; SUVmax: maximum Standardized Uptake Value; SUVmean: mean Standardized Uptake Value; MTV: Metabolic Tumor Volume; TLG: Total Lesion Glycolysis

**Table 3:** A breakdown of FIGO stage migration in 65 patients who were upstaged.

FIGO Stage Post PET					
FIGO stage	IIA	IIB	IIIB	IVA	IVB
	n (%)	n (%)	n (%)	n (%)	n (%)
IB (n = 5)	1 (20.0)	1 (20.0)	0 (0.0)	2 (40.0)	1 (20.0)
IIA (n = 2)	0 (0.0)	0 (0.0)	0 (0.0)	2 (100.0)	0 (0.0)
IIB (n = 20)	0 (0.0)	0 (0.0)	6 (30.0)	5 (25.0)	9 (45.0)
IIIB (n = 37)	0 (0.0)	0 (0.0)	0 (0.0)	16 (43.2)	21 (56.8)
IVA (n = 1)	0 (0.0)	0 (0.0)	0 (0.0)	0 (0.0)	1 (100.0)

### Comparison between HIV-infected and HIV-uninfected patients

The HIV- infected patients were significantly younger compared with HIV-uninfected patients (43.36 ± 8.03 years versus 54.51 ± 13.12, *p*<0.001). More advanced diseases (higher FIGO stages) were seen in HIV-infected patients compared with HIV-uninfected patients (p = 0.022). For example, more than half of HIV-infected patients (56.2%) had a stage III or IV disease compared with 39.6% of HIV-uninfected patients who had a stage III or IV disease. Among HIV-infected women, SCC was more prevalent compared with HIV-uninfected ones (*p* = 0.021). The proportion of patients with regional or distant metastases were not significantly different between the HIV-infected group and HIV-uninfected group. Similarly, the sites of metastases were not significantly different between HIV-infected and HIV-uninfected patients. The proportion of patients who experienced FIGO stage migration were not different between the two groups also. Similarly, FDG-PET metabolic parameters (SUVmax, SUVmean, MTV and TLG) were not significantly different between HIV-infected and HIV-uninfected women. Table 4 shows a detailed comparison between HIV-infected and HIV-uninfected women. Among HIV-infected women, we found no significant difference in the CD4 count or viral load between those women who had regional or distant metastases compared to those with localized disease, p = 0.821 and 0.096 respectively.

**Table 4:** A comparison between the clinical and the FDG-PET/CT image findings between HIV-infected and HIV-uninfected patients.

HIV				
Variable	Positive	Negative	χ^2^	*p*-value
	n (%)	n (%)		
**Age (years)**				
Mean ± SD	43.36 ± 8.03	54.51 ± 13.12	-5.902^t^	<0.001*
**FIGO stage**				
IB	19 (26.0)	8 (15.1)	8.988^Y^	0.022*
IIA	2 (2.7)	4 (7.5)		
IIB	11 (15.1)	20 (37.7)		
IIIB	38 (52.1)	19 (35.8)		
IVA	3 (4.1)	2 (3.8)		
**Histology**				
Squamous cell carcinoma	68 (93.2)	42 (79.2)	5.356	0.021*
Others	5 (6.8)	11 (20.8)		
**Metastasis**				
Yes	51 (69.9)	37 (69.8)	0.000	0.995
No	22 (30.1)	16 (30.2)		
**Site of metastasis (n = 88)**				
Pelvic node only	18 (35.3)	9 (24.3)	2.509^Y^	0.404
Extrapelvic node only	2 (3.9)	3 (8.1)		
Pelvic and extra-pelvic node	14 (27.5)	13 (35.1)		
Visceral only	1 (2.0)	0 (0.0)		
Node and visceral	9 (17.6)	9 (24.3)		
Node and bone	4 (7.8)	0 (0.0)		
Node, visceral and bone	3 (5.9)	3 (8.1)		
**Upstage Post PET**				
Yes	36 (49.3)	29 (54.7)	0.359	0.549
No	37 (50.7)	24 (45.3)		
**SUV max**				
Median (IQR)	18.66 (10.88–26.47)	21.12 (13.07–26.28)	1671.500^U^	0.194
**SUV mean**				
Median (IQR)	5.70 (4.26–7.35)	6.20 (4.48–7.65)	1729.000^U^	0.310
**MTV**				
Median (IQR)	224.35 (71.81–367.36)	177.91 (111.12–268.98)	1825.000^U^	0.588
**TLG**				
Median (IQR)	1355.97 (297.60–2700.47)	1164.06 (543.57–1921.49)	1928.000^U^	0.974

PET: Positron Emission Tomography; SUVmax: maximum Standardized Uptake Value; SUVmean: mean Standardized Uptake Value; MTV: Metabolic Tumor Volume; TLG: Total Lesion Glycolysis; χ^2^: Chi-square test;

^Y^: Yates Corrected Chi-square;

^t^: Independent Samples T-test;

^U^: Mann Whitney U test;

*: *p*-value <0.05;

### The ability of FDG-PET Metabolic Parameters to Predict the Presence of Distant Metastasis

**Fig 1:**
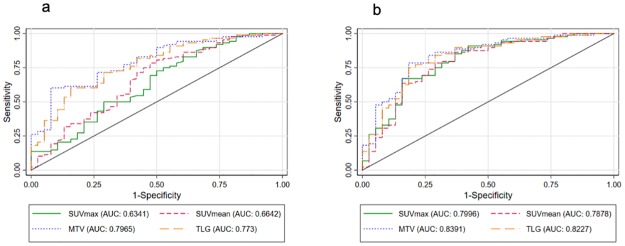
Receiver operating characteristic curves unadjusted (a) and adjusted for FIGO stage of disease post PET/CT imaging (b) showing the performance of FDG-PET metabolic parameters in predicting distant metastasis.

**Fig 2:**
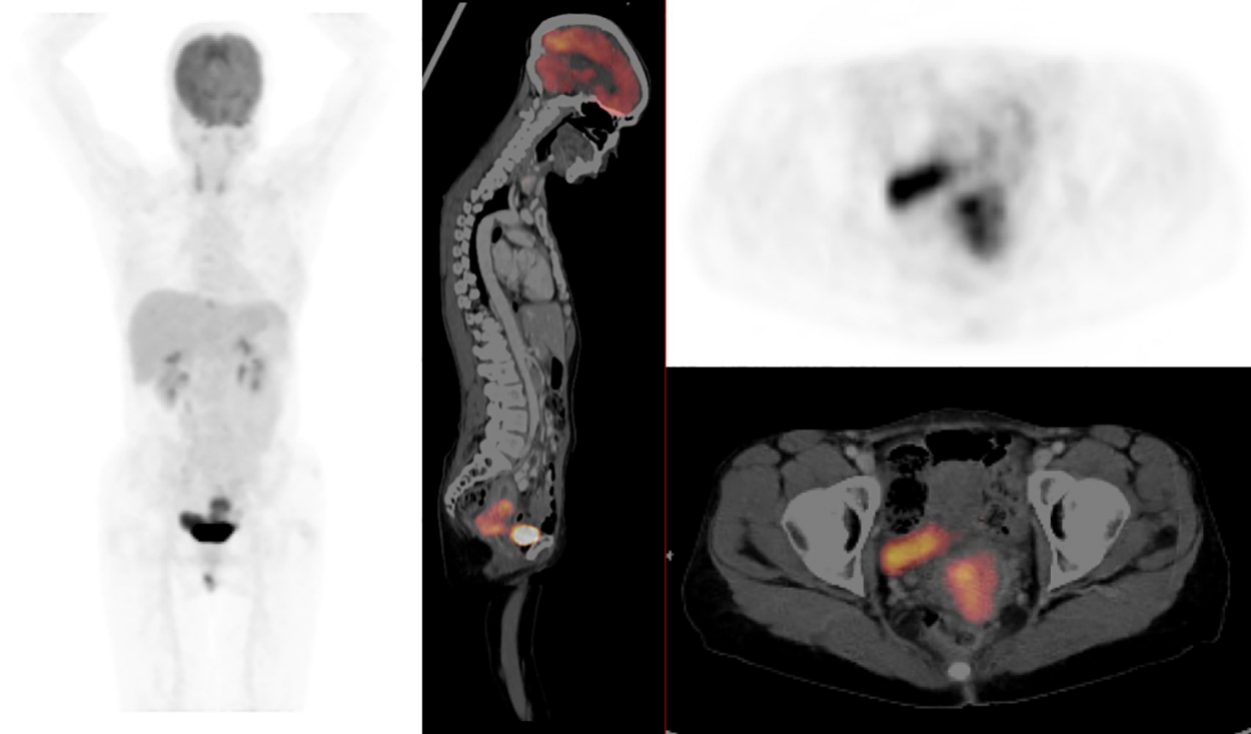
FDG-PET/CT images of a 46-year-old HIV-infected female referred for imaging with stage IIB squamous cell carcinoma of the cervix (SUVmax = 13.06, SUVmean = 4.22, MTV = 186.66, and TLG = 787.77). (A) Coronal PET, (B) sagittal fused PET/CT, (C) axial PET and (D) axial fused PET/CT images show disease localized to the cervix with no distant metastasis.

**Fig 3:**
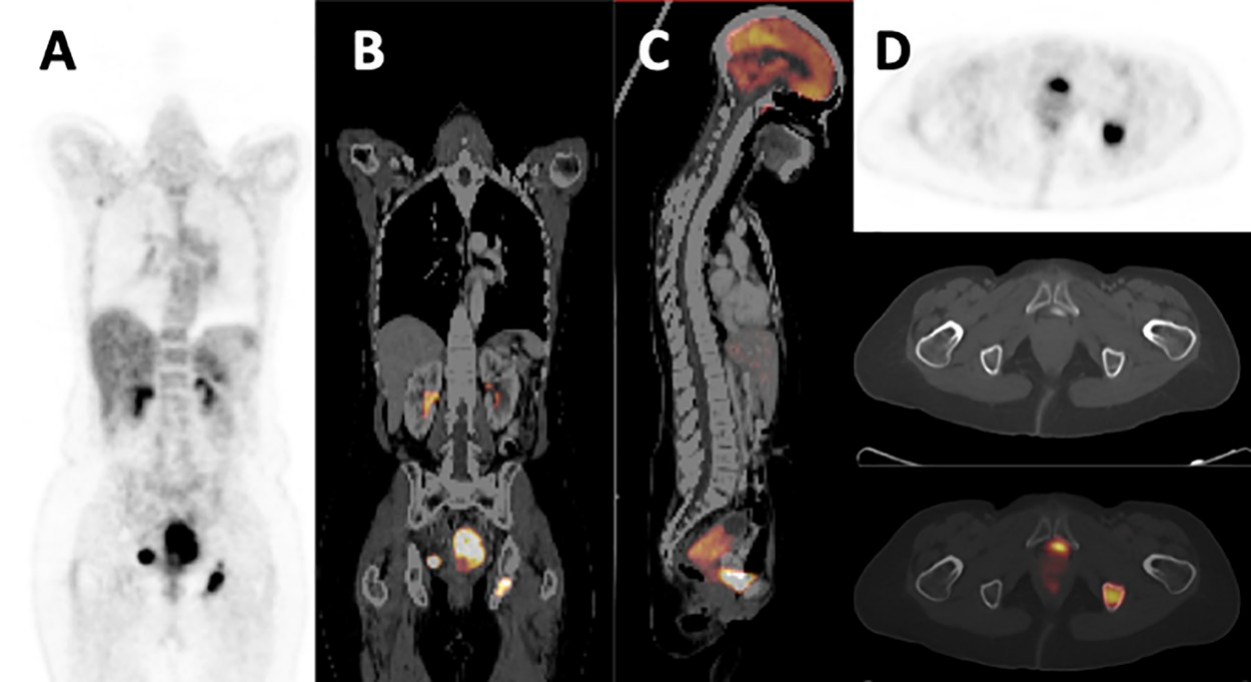
FDG-PET/CT images of a 51-year-old HIV-uninfected female refereed with stage IIIB squamous cell carcinoma of the cervix. (A) Coronal PET, (B) coronal fused PET/CT, (C) sagittal fused PET/CT and (D) axial PET, CT and fused PET/CT images show local disease in the cervix (SUVmax = 24.7, SUVmean = 5.46, MTV = 182.01, and TLG = 993.77) with distant metastasis to right-sided pelvic lymph node and the left ischial bone. She was upstaged to stage IVB disease on account of ischial bone metastasis.

SUVmax, SUVmean, MTV and TLG were all significantly higher among patients who had distant metastases compared with those with localized disease, (*p*<0.05 in all cases), Table 5. The univariable logistic regression showed that these parameters were also all significant in their ability to predict the presence of distant metastasis, Table 6. After we adjusted for the FIGO stage of the disease, only MTV and TLG remained significant predictors of distant metastases. As these parameters increase, the probability of distant metastasis increases, as indicated by the odds ratios being significantly greater than one. Using receiver operating characteristic (ROC) curves, SUVmax, SUVmean, MTV and TLG performed well in predicting the presence of distant metastasis with AUCs of 0.63 (95% CI: 0.52–0.74), 0.66 (95%CI: 0.56–0.77), 0.80 (95%CI: 0.71–0.88) and 0.77 (95%CI: 0.69–0.86) respectively. The performance of these metabolic parameters in predicting distant metastasis improved after we adjusted for the FIGO stage of the disease with AUCs of 0.80 (95%CI: 0.71–0.89), 0.79 (95%CI: 0.69–0.88), 0.84 (95%CI: 0.76–0.92) and 0.82 (95%CI: 0.74–0.91) for SUVmax, SUVmean, MTV and TLG respectively (Fig 1a and 1b). Figs 2 and 3 show images of typical patients we included in this study.

**Table 5:** The difference in FDG-PET metabolic parameters between patients with metastases and those without.

	Metastasis			
Variable	Yes	No	U	*p*-value
	n (%)	n (%)		
**SUV max**				
Median (IQR)	20.69 (12.97–26.67)	14.55 (7.87–24.53)	1223.500	0.017*
**SUV mean**				
Median (IQR)	6.22 (4.93–8.02)	4.72 (3.32–6.79)	1123.000	0.004*
**MTV**				
Median (IQR)	255.12 (129.18–382.03)	81.84 (40.78–187.75)	680.500	<0.001*
**TLG**				
Median (IQR)	1619.36 (607.78–2751.69)	461.44 (153.73–1295.54)	759.000	<0.001*

SUVmax: maximum Standardized Uptake Value; SUVmean: mean Standardized Uptake Value; MTV: Metabolic Tumor Volume; TLG: Total Lesion Glycolysis; U: Mann Whitney U test;

*: *p*-value <0.05

**Table 6:** The ability of FDG-PET metabolic parameters unadjusted and adjusted for the FIGO stage of the disease to predict the presence of distant metastasis.

	Unadjusted logistic regression				Logistic regression adjusted for FIGO stage post PET			
Variable	OR(95%CI)	*p* value	AIC	AUC	OR(95%CI)	*p* value	AIC	AUC
**SUVmax**	1.048(1.009–1.090)	0.016*	151.24	0.63(0.52–0.74)	1.031(0.992–1.0720)	0.121	128.65	0.80(0.71–0.89)
**SUVmean**	1.321(1.083–1.612)	0.006*	149.40	0.66(0.56–0.77)	1.072(0.861–1.337)	0.620	130.89	0.79(0.69–0.88)
**MTV**	1.009(1.005–1.014)	<0.001*	127.81	0.80(0.71–0.88)	1.007(1.002–1.011)	0.003*	119.29	0.84(0.76–0.92)
**TLG**	1.0098(1.00048–1.00147)	<0.001*	134.50	0.77(0.69–0.86)	1.00059(1.00012–1.00107)	0.014*	123.52	0.82(0.74–0.91)

SUVmax: maximum Standardized Uptake Value; SUVmean: mean Standardized Uptake Value; MTV: Metabolic Tumor Volume; TLG: Total Lesion Glycolysis; OR: Odds Ratio; CI: Confidence Interval;

*: *p*-value <0.05

## Discussion

We evaluated the ability of FDG-PET/CT to accurately stage women diagnosed with stages IB to IVA carcinoma of the cervix and found FIGO stage migration to a higher disease state in 51.6% of patients. Patients were upstaged following imaging due to a finding of a more extensive primary tumor than assessed clinically, detection of the urinary bladder or rectal wall invasion or finding of a previously unknown site of distant metastasis. About half of the patients (49.2%) who had stage migration were upstaged to FIGO stage IVB. This stage migration may significantly impact on patients’ management. It must be said however that not all cases of stage migration may lead to a change in treatment option. Patients upstaged to stage IVB due to the detection of a previously unknown sites of distant metastases would definitely have a change in treatment option and intent, from a local/loco-regional treatment to systemic and from a curative intent to palliative treatment intent. Accurate staging of disease is a prerequisite to determining the most suitable therapeutic option. Different stages of carcinoma of the cervix are treated in different ways. Patients with early-stage disease may be offered radical hysterectomy with or without lymphadenectomy followed by adjuvant therapy [12], while patients with locally advanced disease are commonly treated with pelvic radiotherapy with or without concurrent chemotherapy [13,14]. Patients with advanced disease (stage IVB) are treated with single or combination chemotherapy [13]. We demonstrated the sites of metastases in 88 patients with the lymph nodes (pelvic and extra-pelvic) being the most common sites of metastases. The clinical importance of this is that the managing team can decide on the optimum therapy among the available options and as well as guide the field of radiotherapy or extent of surgery.

Among the 126 patients we included in this study, 57.9% had HIV infection. The HIV-infected group was significantly younger and had a higher prevalence of FIGO stage III or IV disease compared to the HIV-uninfected patients. The FDG-PET metabolic parameters, as well as the pattern of distant spread, were not significantly different between the two groups. Similarly, the proportion of patients who experienced stage migration was not significantly different from HIV-infected and HIV-uninfected women suggesting no difference in the impact of FDG-PET/CT for initial staging of disease between the two groups. Detection of a previously unknown site of metastasis in the pelvis such as pelvic lymph node metastasis may lead to a change in radiotherapy field in patients treated by radiotherapy alone or in combination with chemotherapy [15]. These findings provide some guidance on the management of cervical cancer, an acquired immunodeficiency syndrome (AIDS)-defining cancer, among HIV-infected patients. Cancer diagnosis at a younger age means that treatment must be offered while minding the morbidity associated with cancer therapy that may impact on the quality of life after that. Despite a higher prevalence of stage III and stage IV disease among HIV-infected patients, we did not find any significant difference in the FDG-PET metabolic parameters in them versus HIV-uninfected women. Since these parameters have prognostic implications, this may suggest that stage for stage, HIV-infected patients and uninfected patients may be treated in the same way while expecting a similar outcome. This finding is consistent with our findings in other HIV-associated malignancies such as Hodgkin lymphoma and SCC of the anus [16–18]. It also supports the recent recommendation of the National Comprehensive Cancer Network (NCCN) advising that cancers may be treated similarly between HIV-infected and uninfected patients in this current era of effective combination antiretroviral therapy (ART) [19].

We compared the FDG-PET metabolic parameters of the primary tumor between patients with metastases and those without metastasis and found significantly higher values for these parameters in the former compared with the latter group. These parameters reflect the tumor glucose metabolism and hence their aggressiveness [20]. MTV measures the volume of a tumor bulk that is metabolically active. It is, therefore, better at predicting tumor biology than a mere measurement of gross tumor volume that includes areas of necrosis and fibrosis within the tumor bulk. In combination, these parameters have been found to predict response to therapy as well as survival [10,11]. Presence of metastasis, especially to lymph nodes, is a significant predictor of survival in patients with carcinoma of the cervix [11]. All the metabolic parameters we tested in the unadjusted model were significant in predicting the presence of distant metastasis. The probability of metastasis is influenced by the primary tumor size with larger tumors more likely to be associated with distant tumor spread. We, therefore, subsequently adjusted for the FIGO stage of the disease while evaluating the ability of the metabolic parameters for the presence of distant metastasis. Following this adjustment, only MTV and TLG remained significant predictors of distant metastasis. Our findings show that MTV and TLG are more robust in their ability to reflect the tumor behavior compared with SUVmax and SUVmean. Other authors have also reported the correlation between FDG-PET metabolic parameters and nodal metastasis. In a recent study, Zhang et al. found MTV and TLG but not SUVmax to be significant predictors of lymph node metastasis [20]. These workers evaluated only patients with early-stage carcinoma of the cervix (FIGO IB1—IIA) unlike our study with a more heterogeneous patient population. Also, it is unknown if their study population included patients with HIV infection. Chung and colleagues, however, found a significant correlation between SUVmax and nodal metastasis in a group of patients with stage IB to IIA disease [21].

Our study included patients who are typically referred for imaging in cervical cancer staging. We excluded patients with stage IA and IB1 disease as the volume of disease is small in these patients. Small lesions suffer from partial volume averaging leading to an underestimation of the FDG-PET metabolic parameters. We also excluded patients with stage IV disease at the time of referral as these patients were already known with advanced disease and had no significant benefit from imaging.

One of the strengths of our study lies in the unique population we studied, a mix of HIV-infected and HIV-uninfected patients. Even in the current ART era, HIV-associated malignancies remain an increasing cause of mortality among individuals affected with this virus in developed and developing countries alike [22,23]. It is, therefore, necessary to determine the performance of FDG-PET/CT in accurate staging and its metabolic parameters in the determination of the presence of distant metastases among HIV-infected individuals with carcinoma of the cervix.

The results from our results need to be interpreted with bearing in mind the limitations therein. The disease was upstaged in about half of the study population. This high proportion of patients who had stage migration may be as a result of selection bias where patients with suspicion of distant metastasis were more likely to undergo FDG-PET/CT scan compared with patients in whom clinically staging was done with more certainty. Not all lesions seen on FDG-PET/CT were confirmed by histological evaluation as metastatic. This will be impracticable and unethical especially in patients with multiple sites of lesions. Evaluation for distant metastases was based on the histological confirmation, correlative imaging with MRI and the experience of the interpreting physicians. The retrospective design of our study is another limitation. As a result of the retrospective design of this study, we do not have sufficient information on how these patients would have being managed if they had not had FDG-PET/CT imaging preventing us from reporting on the impact of stage migration on patients’ management. A prospectively designed trial confirming our results will be necessary in the future. A phase III trial that compared the role of FDG-PET/CT with stand-alone CT in the initial staging of women with cervical cancer recently reported its findings [24]. The study was underpowered and was terminated due to poor patient recruitment.

## Conclusion

In women with cervical carcinoma, the inclusion of FDG-PET/CT in the pre-therapy assessment improved the accuracy of staging in about of half of patients. FDG-PET metabolic metrics can accurately predict individuals with distant metastases. These metabolic parameters and pattern of metastases are not significantly different between HIV-infected and HIV-uninfected patients.

## Supporting information

S1 Table Supporting information. (XLSX)
